# Rupture of Life‐Threatening Hepatic Artery Pseudoaneurysm After Endoscopic Ultrasonography‐guided Hepaticogastrostomy: Successful Management With Emergency Transcatheter Arterial Embolization

**DOI:** 10.1002/deo2.70176

**Published:** 2025-07-24

**Authors:** Hiroshi Yukimoto, Akino Okamoto, Kohsaku Ohnishi, Keitaro Masuko, Junping Wang, Kazuya Ogawa, Ken Ueda, Motohiro Hirao, Yasuhiro Nakaya, Atsushi Hosui

**Affiliations:** ^1^ Department of Diagnostic Radiology Osaka Rosai Hospital Sakai Osaka Japan; ^2^ Department of Gastroenterology and Hepatology Osaka Rosai Hospital Sakai Osaka Japan

**Keywords:** EUS‐HGS (endoscopic ultrasound‐guided hepatogastrostomy), hepatic artery, plastic stent, pseudoaneurysm, transcatheter arterial embolization

## Abstract

A 70‐year‐old male with lung cancer and interstitial pneumonia was diagnosed with ampullary carcinoma, causing obstructive jaundice. After the failure of endoscopic retrograde cholangiopancreatography, endoscopic ultrasound‐guided hepaticogastrostomy (EUS‐HGS) was performed with a 7‐Fr plastic stent (PS) into the B2 bile duct. Three months later, mild bleeding was observed during stent exchange, but was stopped by stent replacement. The patient developed recurrent cholangitis, and 1 month later, when the PS was removed to add supplementary drainage, massive bleeding occurred from the endosonographically created route into the stomach. Contrast‐enhanced computed tomography (CECT) revealed a pseudoaneurysm in the A2 branch of the hepatic artery. Emergency angiography confirmed active extravasation, and successful transcatheter arterial embolization with *N*‐butyl‐2‐cyanoacrylate was performed. The patient recovered without rebleeding but died two weeks later from worsening interstitial pneumonia. A review of publications identified only three previous cases of pseudoaneurysm after EUS‐HGS, all of which involved self‐expandable metal stents. This case demonstrates that pseudoaneurysms can cause both gastrointestinal bleeding and recurrent cholangitis. Careful evaluation of CECT images is needed before stent manipulation in patients with biliary symptoms after EUS‐HGS.

## 1 Introduction

Endoscopic ultrasound‐guided hepaticogastrostomy (EUS‐HGS) has emerged as an alternative biliary drainage technique for patients with malignant biliary obstruction when conventional endoscopic retrograde cholangiopancreatography (ERCP) is unsuccessful or not feasible. Since its initial introduction, EUS‐HGS has gained increasing acceptance for managing obstructive jaundice in patients with altered anatomy or duodenal obstruction due to tumor invasion [1].

While EUS‐HGS is generally considered safe and effective, it carries potential risks of adverse events, including bleeding, bile leakage, peritonitis, pneumoperitoneum, and stent migration. The incidence of bleeding after EUS‐HGS was reported to be 3.7% [2], but most cases are related to immediate procedural complications rather than delayed vascular events.

Hepatic artery (HA) pseudoaneurysms after EUS‐HGS procedures are concerned with severe problems as they may remain clinically silent until rupture occurs, at which point hemobilia, gastrointestinal bleeding, or hemorrhagic shock is observed. The pathophysiological analysis reveals that direct vascular injury, mechanical irritation from the stent, or erosion against adjacent vessels over time are involved in the development of pseudoaneurysms. Prompt detection and appropriate management are crucial for successful outcomes [3, 4].

We present a case of delayed rupture of an HA pseudoaneurysm after EUS‐HGS in a patient with ampullary carcinoma, describing the clinical presentation, diagnostic approach, and successful management with transcatheter arterial embolization (TAE).

### 1.1 Case Report

**FIGURE 1:**
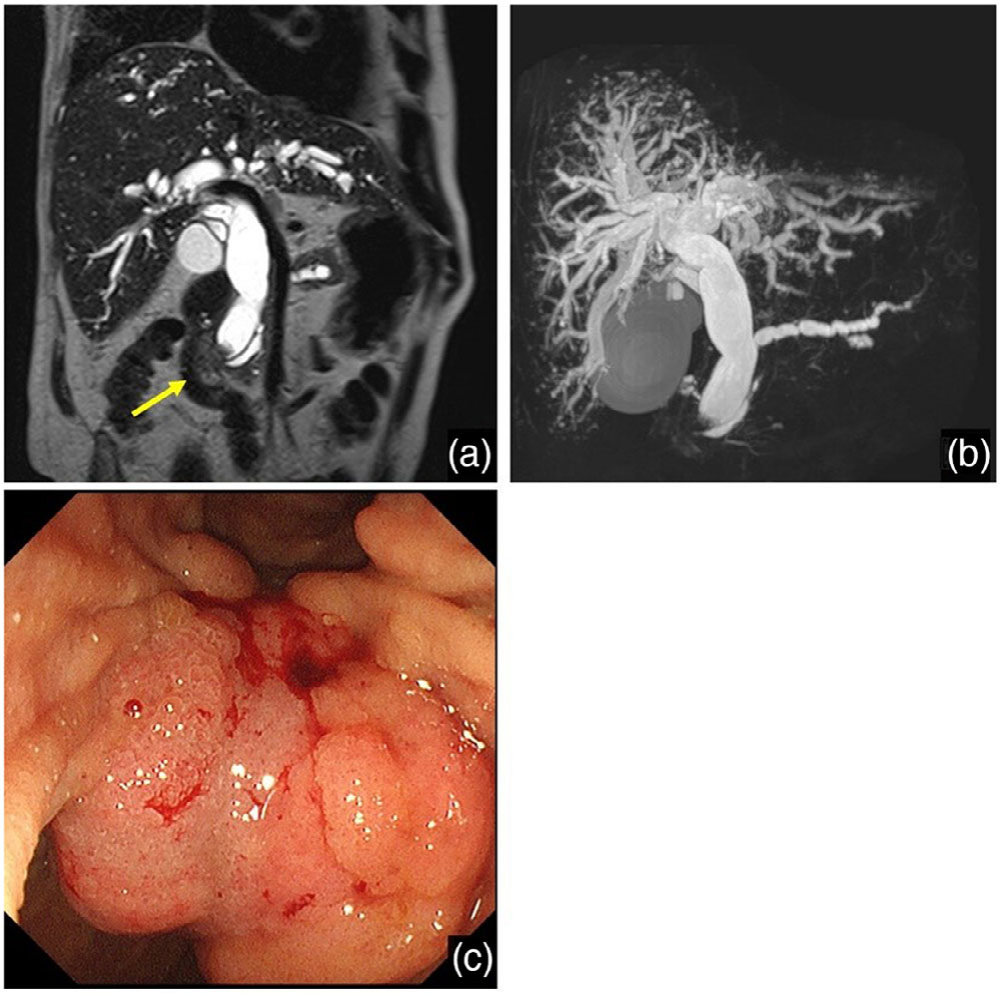
Imaging findings of ampullary carcinoma. (A) T2‐weighted magnetic resonance imaging (MRI) showing a tumor at the ampulla of Vater (yellow arrow) with biliary and pancreatic duct dilation. (B) Magnetic resonance cholangiopancreatography (MRCP) demonstrating dilation of the biliary tree due to ampullary obstruction. (C) Endoscopic view of the large ampullary tumor obscuring the biliary orifice, which prevented successful ERCP.

A 70‐year‐old male patient with lung cancer and interstitial pneumonia was diagnosed with ampullary carcinoma. Lung cancer had been previously treated with pembrolizumab, which was discontinued due to the exacerbation of interstitial pneumonia. Five milligrams of prednisolone was treated for the interstitial pneumonia. During this treatment, jaundice appeared and worsened, and he was referred to our hospital. Blood tests showed elevated hepatobiliary enzymes and carcinoembryonic antigen (9.2 ng/mL). Contrast‐enhanced computed tomography (CECT) and magnetic resonance imaging (MRI) revealed a tumor at the ampulla of Vater with dilation of the biliary and pancreatic ducts (Figures 1A,B). Enlarged lymph nodes were observed around the tumor and in the paraaortic region. A biopsy of the tumor allowed diagnosis of adenocarcinoma (cT2N1M1; Stage IV according to UICC eighth edition).

**FIGURE 2:**
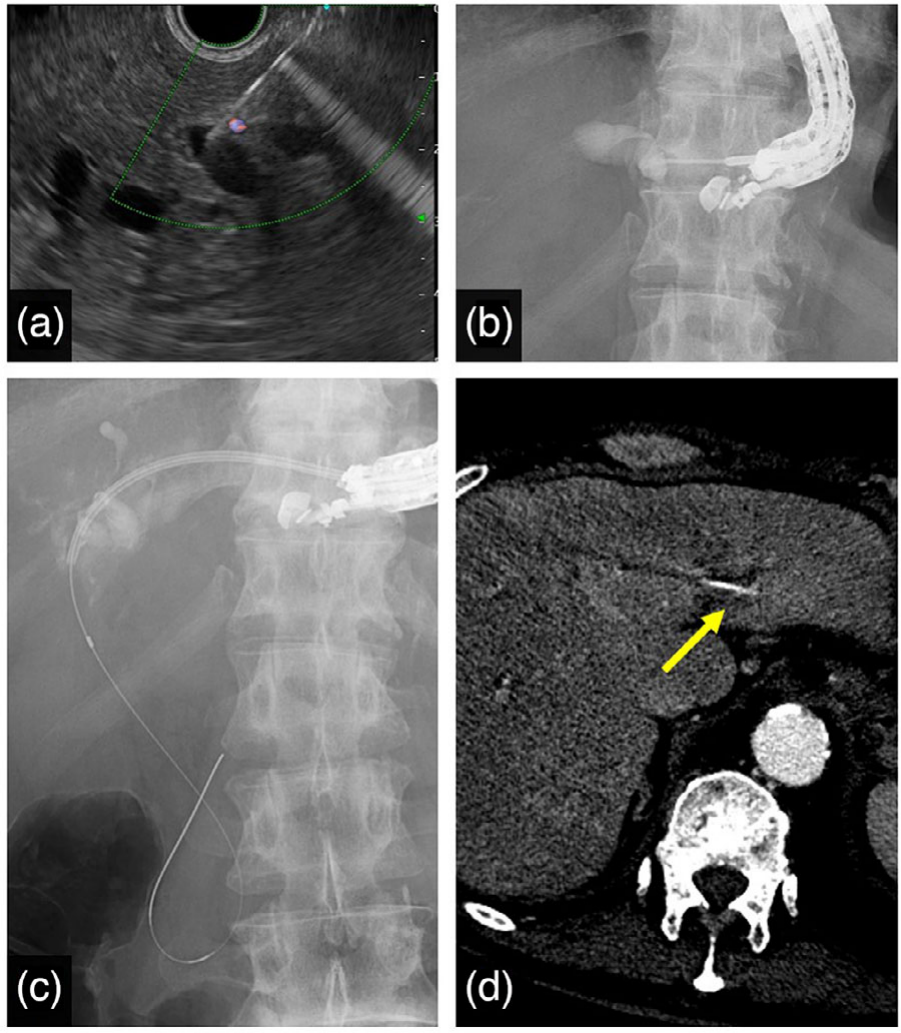
Endoscopic ultrasound‐guided hepaticogastrostomy (EUS‐HGS) procedure. (A) EUS image showing puncture of the dilated B2 bile duct with color Doppler to avoid vascular structures. (B) Cholangiography after successful bile duct puncture. (C) The 7‐Fr plastic stent (PS) successfully deployed through the EUS‐HGS route. (D) Post‐procedural CECT demonstrating the close spatial relationship between the deployed PS and the A2 branch of the HA (yellow arrow), which predisposed to subsequent vascular injury.

Due to obstructive jaundice, ERCP was attempted, but the biliary orifice was not identified because of tumor invasion (Figure 1C). EUS‐HGS was performed the next day. Under the guidance of EUS, vascular structures were carefully avoided, and the dilated 7 mm B2 bile duct was punctured with a 19‐gauge needle (EZ‐Shot‐3Plus; Olympus Corporation, Tokyo, Japan) (Figure 2A). After contrast administration, a 0.025‐inch guidewire (Visiglide2; Olympus Corporation) was placed into the common bile duct on the first attempt (Figure 2B). Bile was aspirated with a 7.2‐Fr double lumen catheter (Uneven Double Lumen Catheter; PIOLAX Medical Devices Inc., Yokohama, Japan). Without additional tract dilation, a 7‐Fr, 14 cm plastic stent (PS) (Through Pass Type IT; Gadelius Medical, Tokyo, Japan) was placed (Figure 2C). Twenty days later, jaundice had improved, and chemotherapy with S‐1 was started.

**FIGURE 3:**
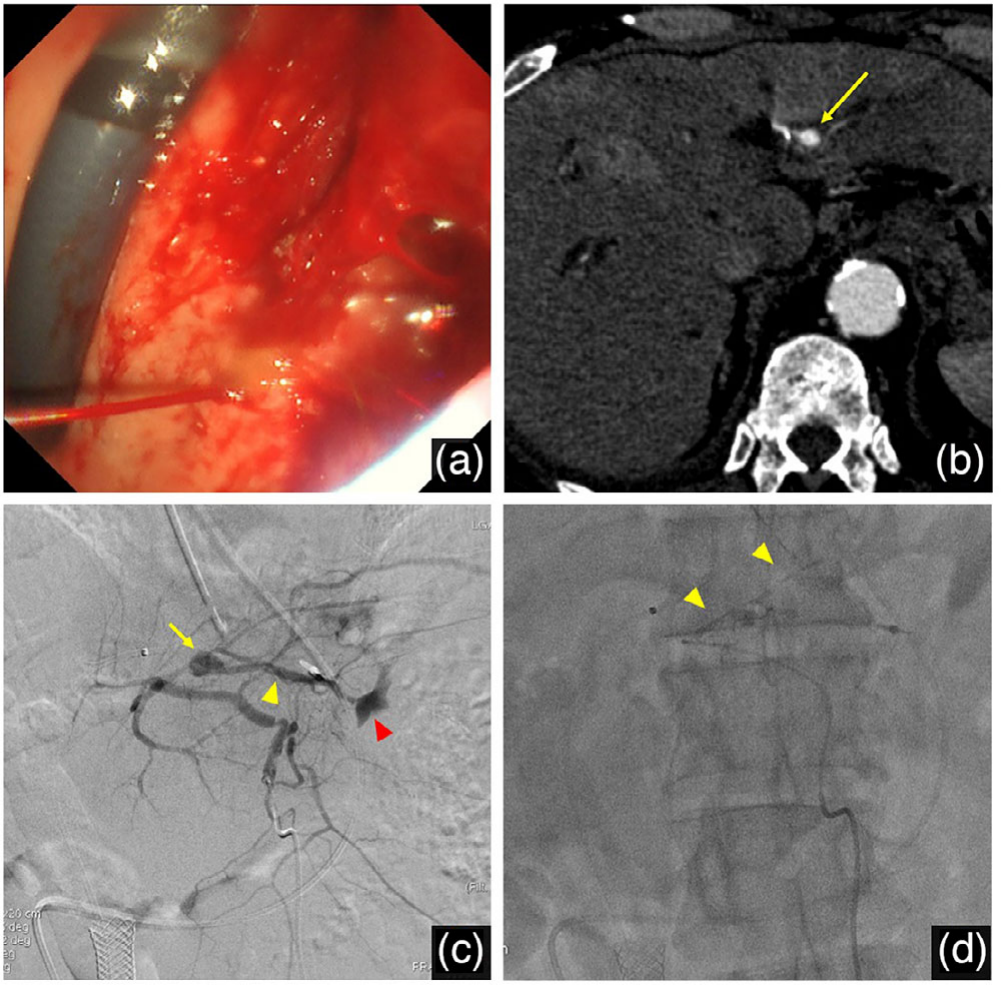
Bleeding complications and management. (A) Endoscopic view showing massive spouting bleeding from the endosonographically created route (ESCR) into the stomach. (B) Contrast‐enhanced computed tomography (CECT) scan revealing an 8 mm pseudoaneurysm (yellow arrow) in the A2 branch of the hepatic artery adjacent to the ESCR. (C) Angiography confirming a pseudoaneurysm (yellow arrow) with contrast extravasation from the ESCR (yellow arrowhead) flowing into the stomach (red arrowhead). (D) Post‐embolization angiography showing complete occlusion with *N*‐butyl‐2 cyanoacrylate (NBCA) cast formation (yellow arrowheads).

Three months later, during a scheduled stent exchange, mild bleeding was observed from the endosonographically created route (ESCR) after removing the PS. Hemostasis was temporarily achieved by placing another PS of the same specifications. After discharge, the patient experienced two episodes of cholangitis over the subsequent month, requiring antibiotic treatment and leading to the decision for readmission to add antegrade transpapillary drainage through the ESCR. When the PS was removed, massive spouting bleeding occurred from the ESCR (Figure 3A). Poor visualization and limited scope maneuverability due to ongoing bleeding made it impossible to achieve endoscopic hemostasis and to replace a PS through the ESCR. A 10 mm × 6 cm fully‐covered self‐expandable metal stent (SEMS) (WallFlex; Boston Scientific, Natick, MA, USA) was successfully placed retrogradely, which had become accessible as the primary tumor had decreased in size by chemotherapy. An endoscopic nasobiliary drainage (ENBD) tube was also set to ensure reliable biliary drainage for cholangitis treatment and to monitor bleeding. The bleeding episode and subsequent management are documented in the supplementary video (Video S1).

A review of CECT scans revealed the development of an 8 mm pseudoaneurysm in the A2 branch of the HA adjacent to the ESCR after the previous stent exchange (Figures 2D and 3B). The patient went into shock due to bleeding, and emergent TAE was performed because massive contrast extravasation was found from the pseudoaneurysm into the stomach via the ESCR by angiography (Figure 3C). Although isolation of the pseudoaneurysm was initially attempted with micro‐coils, selective catheterization of the small distal artery from within the large pseudoaneurysm was technically challenging. Given the patient's hemodynamic instability, we performed embolization using *N*‐butyl‐2 cyanoacrylate (NBCA) mixed with Lipiodol in a 1:3 ratio (total injection volume 0.3 mL), which was injected with controlled catheter withdrawal to achieve complete cast‐like occlusion from distal to proximal portions of the pseudoaneurysm (Figure 3D). Complete hemostasis was achieved without complications.

After TAE, liver enzymes showed mild transient elevations (aspartate aminotransferase from 44 to 98 IU/L, alanine aminotransferase from 30 to 49 IU/L) which normalized within several days, though it was unclear whether this was related to cholangitis or the TAE procedure. Unfortunately, two weeks later, the patient died from worsening interstitial pneumonia. While the major bleeding episode and subsequent procedures may have contributed to overall deterioration, no recurrent bleeding or significant TAE‐related complications were observed.

## 2 Discussion

In reviewing the available literature, we identified only three previous reports about the formation of pseudoaneurysms after EUS‐HGS (Table 1). Prachayakul et al. attributed pseudoaneurysm to the use of a Soehendra stent retriever during fistula dilation [3]. Yamada et al. described late‐onset pseudoaneurysm (50 days post‐procedure) caused by mechanical factors such as gastric peristalsis, stent radial force, and axial force, in addition to inflammation from cholangitis [4]. Ban et al. reported a pseudoaneurysm 60 days after EUS‐HGS with a partially covered SEMS, suggesting drill dilator‐related arterial damage [5].

**TABLE 1:** Published case reports of hepatic artery pseudoaneurysm after endoscopic ultrasound‐guided hepaticogastrostomy (EUS‐HGS).

Author	Year	Age	Sex	Disease	Needle	Dilation	Stent	Artery	Location of the stent	Symptoms	Interval	Treatment	Rebleeding
Prachayakul ^[^ ^3^ ^]^	2013	66	M	PDAC	19G	7‐Fr: SSR 8.5/10‐Fr: dilator	10‐mm FC‐SEMS	LHA	Middle	Melena	14	TAE (Coils)	None
Yamada^[^ ^4^ ^]^	2023	65	M	PDAC	19/22G	7‐Fr: Tornus 6‐mm: balloon	8‐mm PC‐SEMS	LHA	Hepatic edge	Anemia cholangitis	50	TAE (Coils)	None
Ban^[^ ^5^ ^]^	2024	80	F	PDAC	19G	7‐Fr: Tornus 4‐mm: balloon	8‐mm PC‐SEMS	LHA	Middle	Hematemesis cholangitis	60	TAE (Coils)	None
Current case	2025	70	M	AC	19G	7.2Fr: Catheter	7‐Fr. PS	LHA	Middle	Cholangitis	132	TAE (NBCA)	None

Abbreviations: AC, ampullary carcinoma; F, female; FC‐SEMS, fully covered self‐expandable metal stent; LHA, left hepatic artery; M, male; PC‐SEMS, partially covered self‐expandable metal stent; PDAC, pancreatic ductal adenocarcinoma; PS, plastic stent; SSR, Soehendra stent retriever; TAE, transcatheter arterial embolization.

Interestingly, all three previously reported cases involved SEMS, whereas our case occurred with a PS. PSs generally exert less axial and radial force than SEMS, and our puncture site in the upper stomach would typically have less effect from gastric peristalsis. Considering that the pseudoaneurysm appeared after the initial stent exchange and the initial bleeding episode occurred during this exchange, we suspect that the injury to HA was likely caused by the PS flap damaging the arterial wall during the exchange procedure, which eventually led to the formation of the pseudoaneurysm.

It is clinically important that pseudoaneurysms after EUS‐HGS can manifest not only as gastrointestinal bleeding but also as recurrent cholangitis. Careful evaluation of CECT images is recommended for potential pseudoaneurysms in patients with recurrent cholangitis after EUS‐HGS, particularly when stent exchange or removal is planned. In our case, the initial mild bleeding was endoscopically controlled with stent replacement, and hemoglobin levels remained stable, leading us to suspect mucosal rather than vascular injury. However, in retrospect, CECT should have been performed after any bleeding episode to evaluate for potential vascular complications, representing an important clinical learning point.

Regarding treatment, coil embolization is typically preferred for management of HA pseudoaneurysm as it allows for precise isolation of the pseudoaneurysm, providing precise control and limited hepatic ischemia [6, 7]. However, in our case, the technical difficulty of selecting the distal artery and the urgent need for hemostasis led us to choose embolization with NBCA. The use of NBCA offers the advantage of providing rapid hemostasis when catheterization of the pseudoaneurysm or its distal branches is technically challenging [8], and in patients with coagulation abnormalities [9], although it requires careful adjustment of NBCA concentration and technical expertise to avoid non‐target embolization.

HA pseudoaneurysm is an extremely rare but potentially life‐threatening complication of EUS‐HGS. Key clinical lessons include: (1) pseudoaneurysms may present as recurrent cholangitis rather than overt bleeding; (2) CECT imaging should be considered before stent manipulation in patients with post‐EUS‐HGS biliary symptoms; and (3) even PSs can contribute to delayed vascular complications. TAE remains the optimal treatment, with NBCA providing effective, rapid hemostasis in emergency situations. As EUS‐HGS becomes more common, awareness of this complication and established protocols for its management will be essential for optimal patient outcomes.

## Conflicts of Interest

The authors declare no conflicts of interest.

## Consent

Informed consent for publication, including radiologic and endoscopic images, was obtained from the patient's family.

## Supporting information




**VIDEO S1** Video documentation of bleeding episode during EUS‐HGS stent exchange and subsequent management.
